# Unveiling the novel immune and molecular signatures of ovarian cancer: insights and innovations from single-cell sequencing

**DOI:** 10.3389/fimmu.2023.1288027

**Published:** 2023-11-01

**Authors:** Zhongkang Li, Haihan Gu, Xiaotong Xu, Yanpeng Tian, Xianghua Huang, Yanfang Du

**Affiliations:** ^1^ Department of Obstetrics and Gynecology, The Second Hospital of Hebei Medical University, Shijiazhuang, Hebei, China; ^2^ Department of Pharmacy, The Second Hospital of Hebei Medical University, Shijiazhuang, Hebei, China; ^3^ Department of Obstetrics and Gynecology, The First Affiliated Hospital of Zhengzhou University, Zhengzhou, Henan, China

**Keywords:** ovarian cancer, single-cell sequencing, tumor immunology, tumor heterogeneity, tumor microenvironment, transcriptomics, multi-omics, spatial transcriptomics

## Abstract

Ovarian cancer is a highly heterogeneous and lethal malignancy with limited treatment options. Over the past decade, single-cell sequencing has emerged as an advanced biological technology capable of decoding the landscape of ovarian cancer at the single-cell resolution. It operates at the level of genes, transcriptomes, proteins, epigenomes, and metabolisms, providing detailed information that is distinct from bulk sequencing methods, which only offer average data for specific lesions. Single-cell sequencing technology provides detailed insights into the immune and molecular mechanisms underlying tumor occurrence, development, drug resistance, and immune escape. These insights can guide the development of innovative diagnostic markers, therapeutic strategies, and prognostic indicators. Overall, this review provides a comprehensive summary of the diverse applications of single-cell sequencing in ovarian cancer. It encompasses the identification and characterization of novel cell subpopulations, the elucidation of tumor heterogeneity, the investigation of the tumor microenvironment, the analysis of mechanisms underlying metastasis, and the integration of innovative approaches such as organoid models and multi-omics analysis.

## 1 Introduction

Ovarian cancer (OC) is a highly lethal malignancy affecting the female reproductive system with high mortality rates worldwide. It is often diagnosed during late stages, given that early symptoms are often absent and effective screening methods are lacking (1). Despite significant advancements in cytoreductive surgery, chemotherapy, immunotherapy, and maintenance therapy, the 5-year survival rate for OC remains discouraging, with reported rates of 26% to 42% (2, 3). This is mainly attributed to drug resistance, recurrence, and metastasis, which are commonly observed in OC cases (4). Additionally, the substantial heterogeneity and complex molecular properties of ovary-derived carcinoma present challenges in understanding its origins and progression. Consequently, comprehensive exploration of crucial biomarkers and molecular mechanisms in OC is imperative for early diagnosis, innovating therapeutic strategies, and accurately predicting prognosis.

In recent years, bulk RNA sequencing (RNA-seq) technology has been widely utilized to investigate the molecular characteristics and biological processes of OC (5). However, the complexity of the tumor microenvironment (TME) presents a formidable challenge when using bulk RNA-seq, as it includes malignant cells and various other functional cells. Consequently, bulk transcriptomics is unsuitable for cell-level research on tumor tissue characterized by high heterogeneity and complex components (6). This limitation hinders the acquisition of a precise understanding of the underlying biological and pathological processes. Over the past decade, single-cell sequencing has emerged as a potent biological technology capable of decoding the landscape of OC at the level of genes, transcriptomes, proteins, epigenomes, and metabolisms level (7). Specifically, Single-cell sequencing provides valuable insights into the intra- and inter-tumor heterogeneity, enabling the analysis of cellular variations, identification of cell classifications, and comprehension of carcinogenesis to accelerate the development of novel and efficient therapies (8). Single-cell sequencing technology has been widely utilized in OC research, providing significant insights into this complex disease. For instance, single-cell RNA sequencing (scRNA-seq) data analysis has indicated that targeting the JAK/STAT pathway may hold anti-tumor efficacy in OC (9). Similarly, using scRNA-seq, researchers identified six subtypes of fallopian tube epithelium (FTE), with one subtype being associated with clinical prognosis (10).

In this review, we expound on the utilization of single-cell sequencing in exploring cell types, elucidating tumor heterogeneity, uncovering the tumor microenvironment landscape, and analyzing the mechanism underlying OC metastasis. Furthermore, we discuss its integration with novel methods, including organoid culture and multi-omics analysis. The utilization of single-cell sequencing technology provides a wealth of detailed insights into the molecular mechanisms involved in tumor initiation, progression, drug resistance, and immune evasion. These discoveries will provide valuable guidance for the development of innovative therapeutic strategies, the identification of diagnostic markers, and the prediction of patient prognosis.

## 2 Epidemiological and biological characteristics of OC

In 2020, the global incidence of OC exceeded 300,000, with more than 200,000 reported deaths (11). Ovarian malignancies are frequently diagnosed at advanced stages due to the lack of reliable early detection methods, resulting in poor prognosis and ineffective therapeutic targets (12). Furthermore, OC can be classified into over fifteen distinct molecular and pathological subtypes, making it challenging to determine the optimal treatment strategies (13, 14). Ovarian epithelial carcinomas are the predominant and deadliest forms of OC. Ovarian carcinomas are categorized into five primary types based on histopathology and molecular genetic alterations, including high-grade serous (70%), endometrioid (10%), clear cell (10%), mucinous (3%), and low-grade serous (less than 5%) (15). Among these types, high-grade serous ovarian cancer (HGSOC) accounts for approximately 80% of OC-related deaths (16).

Cytoreductive surgery and platinum-based chemotherapy currently serve as the primary treatments for ovarian carcinoma. The past few years have witnessed the advent of targeted therapies that focus on the unique molecular characteristics of OC and play an increasingly important role. Specifically, approximately 20% of HGSOC cases exhibit alterations in BRCA1/2 genes, either through mutations or methylation modifications (17). Mutations in BRCA1/2 are widely acknowledged to be a significant contributing factor to homologous recombination deficiency (HRD) in ovarian carcinoma. Cells with these mutations rely on error-prone DNA repair mechanisms, making them susceptible to DNA damaging agents and poly (ADP-ribose) polymerase inhibitors (PARPi) (18). Additionally, anti-angiogenic agents targeting the VEGF/VEGFR signaling pathway in the TME have been developed for OC (19). The emergence of this innovative targeted approach has created opportunities for stratifying OC patients and providing personalized treatment approaches (14). However, due to the high intra-tumor heterogeneity, nearly 50% of OC patients without an HR mutation exhibit platinum resistance or refractoriness, and most treated patients will eventually experience tumor relapse and metastasis (20, 21). Furthermore, relapsed patients often develop drug resistance, experience severe chemotherapy side effects, and eventually succumb to the disease (2). Therefore, the identifying novel molecular targets and the implementing personalized therapy are crucial for improving the survival rate of OC patients.

An increasing body of evidence from multi-omics studies recognizes OC as a distinct disease characterized by significant heterogeneity resulting from various molecular and microenvironmental factors (17, 22). Nonetheless, lacking a comprehensive understanding of the TME in OC has hindered the advancement of novel immunotherapy approaches and classification methods. Gaining insight into gene expression and mutations is crucial for assessing patient prognosis and evaluating the efficacy of chemotherapy (23, 24). The key challenges in understanding ovarian tumors lie in detecting rare cancer cell subtypes with genetic variability and elucidating the role of stromal cells in providing functional support, which can contribute to chemoresistance. A meta-analysis study analyzing a large amount of bulk sequencing data in OC failed to identify a satisfactory predictive gene expression signature (25). Despite extensive analyses, no practical and novel biomarkers have been identified for prognosis and immunotherapy. Consequently, genetic and molecular studies conducted at the bulk tissue level have not obtained significant clinical insights. Interestingly, the emergence of single-cell sequencing techniques has facilitated the classification of individual cells based on their subtypes and expanded our understandings of the molecular characteristics of ovarian tumors (26). Single-cell sequencing methods provide a comprehensive assessment of intra-tumor heterogeneity in OC by enabling high-resolution molecular profiling of numerous distinct malignant cells and stromal and immune-associated cells within the tumor. By conducting single-cell resolution analysis of tumor tissue with complex compositions, researchers can comprehensively depict the intra-tumor and inter-patient heterogeneity while simultaneously capturing the quantitative and qualitative aspects of thousands of genes at the single-cell level.

## 3 Emerging single-cell sequencing technology

The emergence of next-generation sequencing technology in 2005 marked a significant milestone in the rapid development of biological science (27). High-throughput sequencing of genes, transcriptomes, and non-coding RNA using next-generation sequencing has paved the way for single-cell sequencing. Tang et al. conducted the first single-cell transcriptomics investigation in 2009, analyzing mouse blastomeres, although it was considered “low throughput” (28). The first DNA sequencing of malignant tumor cells at a single-cell resolution was accomplished in 2011 (29). Over the past decade, single-cell sequencing has rapidly developed, stemming from the advancements in next-generation sequencing.

The past few years have witnessed unprecedented progress in analyzing intra- and inter-tumor cellular heterogeneity and the biological processes of tumor development at a high resolution using single-cell sequencing, outperforming previous traditional sequencing methods. It also enhanced researchers’ ability to investigate molecular features in different cell types, ensuring unbiased analysis of cancer tissue (30, 31). Furthermore, it enabled the detection of single-nucleotide variations and copy number alterations at the molecular level (32, 33). Single-cell analysis of multiple omics, including gene sequencing, transcriptome analysis, protein profiling, epigenomic characterization, and metabolic profiling, has experienced significant advancements in recent years (34). Innovations in cell separation/isolation techniques, sequencing technologies, and analytical procedures have propelled the advancement of single-cell sequencing, enhancing its accuracy and sensitivity. Consequently, this acceleration has facilitated the advancement of effective treatments and personalized therapies by constructing of human cell atlases and further cancer research (35, 36). In cancer research, single-cell analysis has been employed to identify and characterize rare cellular subtypes, cancer stem cells (CSCs), circulating tumor cells (CTCs), TME, inter-tumor heterogeneity, and molecular mutations (37, 38). These detailed insights into the molecular mechanisms underlying tumor initiation, progression, metastasis, drug resistance, recurrence, and immune evasion, provide valuable guidance for cancer research.

**Figure 1:**
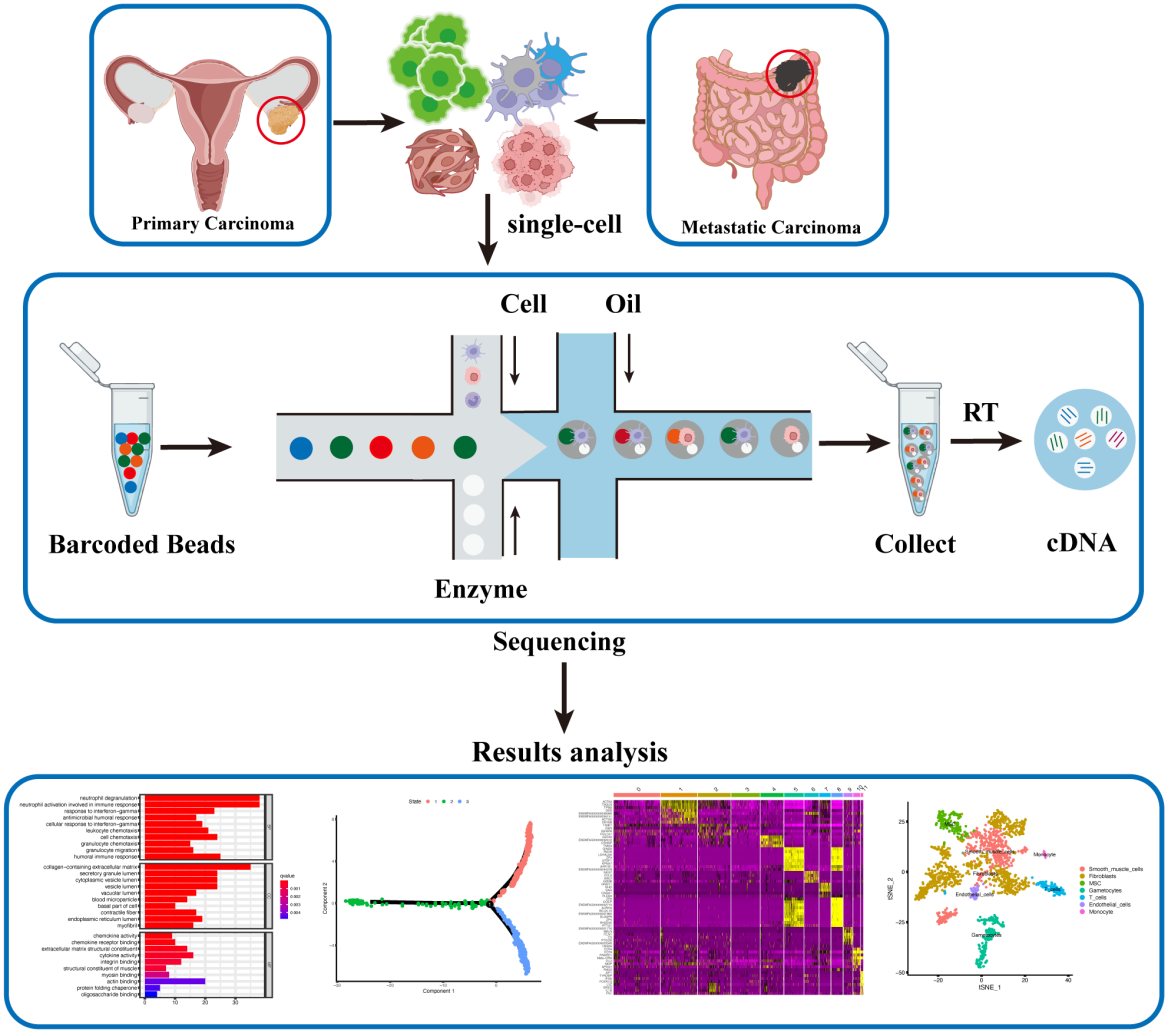
Single-cell Sequencing Workflow for Ovarian Cancer. Single-cell suspensions are isolated from fresh ovarian cancer and metastatic cancer tissues. Barcoded Beads labeling technology is employed to obtain gene expression levels and profiles for each individual cell post-sequencing. Subsequent analysis, including cell clustering, differential gene expression, cell developmental trajectory, and differential gene clustering, generates diverse datasets, offering a comprehensive and updated understanding of ovarian cancer at the single-cell level.

scRNA-seq is a widely employed method for profiling the transcriptomes of individual cells, making it one of the most utilized technologies in this field. The Drop-seq-based platform developed by 10X Genomics (39) and the CytoSeq-based platform offered by BD Rhapsody (40) are widely utilized for scRNA-seq experiments. Furthermore, the other key single-cell sequencing technologies and platforms have been compiled in **Table 1**. The scRNA-seq follows general protocols, which involve isolating single cells, extracting RNA, performing reverse transcription, conducting detection, and finally, carrying out bioinformatic analyses (41) (**Figure 1**).

**Table 1:** Summary of single-cell sequencing technologies and platforms.

Technology	Platform	Year	Single-cell Isolation Method	Barcode Type	Library Preparation Method	Throughput
**Transcriptomics**	SMART-seq2	2013	FACS	Unique Molecular Identifier	Full-length cDNA amplification	Low
	Drop-seq	2015	Droplet-based microfluidics	Unique Molecular Identifier	cDNA amplification	High
	inDrop	2015	Droplet-based microfluidics	Unique Molecular Identifier	cDNA amplification	High
	10x Genomics Chromium	2015	Microfluidics, FACS	Unique Molecular Identifier	cDNA amplification	High
	CytoSeq	2015	microwells	Unique Molecular Identifier	cDNA amplification	High
	Seq-Well	2017	Microfluidics	Unique Molecular Identifier	cDNA amplification	High
	MARS-seq	2017	Microfluidics	Unique Molecular Identifier	cDNA amplification	High
	sci-RNA-seq2	2019	Microfluidics	Unique Molecular Identifier	cDNA amplification	High
	CEL-Seq2	2015	Micromanipulation, FACS	Unique Molecular Identifier	cDNA amplification	Low
	Quartz-Seq	2017	Microfluidics	Unique Molecular Identifier	cDNA amplification	High
	SPLiT-seq	2017	Micromanipulation, FACS	Unique Molecular Identifier	cDNA amplification	High
**Genomics**	10x Genomics Chromium	2015	Microfluidics, FACS	Unique Molecular Identifier	Genome amplification	High
	MARS-seq	2017	Microfluidics	Unique Molecular Identifier	Genome amplification	High
	Seq-Well	2017	Microfluidics	Unique Molecular Identifier	Genome amplification	High
	sci-RNA-seq2	2019	Microfluidics	Unique Molecular Identifier	Genome amplification	High
	CEL-Seq2	2015	Micromanipulation, FACS	Unique Molecular Identifier	Genome amplification	Low
	SPLiT-seq	2017	Micromanipulation, FACS	Unique Molecular Identifier	Genome amplification	High
**Epigenomics**	scATAC-seq	2015	Tn5 tagmentation	Unique Molecular Identifier or ATAC-barcode	Genome amplification/tagmentation	High
	scCHIC-seq	2017	Tn5 tagmentation	Unique Molecular Identifier or ATAC-barcode	Genome amplification/tagmentation	High
	scNMT-seq	2017	Tn5 tagmentation	Unique Molecular Identifier	Genome amplification	High
	scCOOL-seq	2019	Tn5 tagmentation	Unique Molecular Identifier	Genome amplification	High
	scTHS-seq	2020	Tn5 tagmentation	Unique Molecular Identifier	Genome amplification	High
	scMC-seq	2020	Tn5 tagmentation	Unique Molecular Identifier	Genome amplification	High
**Spatial Transcriptomics**	10x Genomics Visium	2016	Spatial capture, *in situ* sequencing	Spatial barcode	cDNA amplification	High
	Slide-seq	2020	Spatial capture, *in situ* sequencing	Spatial barcode	cDNA amplification	High
	MERFISH	2016	Single-molecule FISH	Oligonucleotide probes	Imaging	
	seqFISH+	2017	Single-molecule FISH	Oligonucleotide probes	Imaging	
	osmFISH	2019	Single-molecule FISH	Oligonucleotide probes	Imaging	
	STARmap	2019	Single-molecule FISH	Oligonucleotide probes	Imaging	
	FISSEQ	2019	Single-molecule FISH	Oligonucleotide probes	Imaging	

Single-cell isolation is the initial and crucial step that determines the accuracy and sensitivity of single-cell sequencing. The primary challenge lies in the rapid and precise collection of these individual cells. Experimental tissues are typically obtained during cytoreductive surgery after pathological diagnosis, and various methods are employed to isolate an adequate number of single cells. The original protocol used Flow Activated Cell Sorting (FACS) to isolate single cells (42). Modern techniques, such as Drop-seq and SCI-seq, utilize microfluidics with droplets and nanowells to achieve high-throughput single-cell separation, thereby enhancing sequencing efficiency and ease (39, 43, 44). Given that the preparation of single-cell suspensions requires fresh samples, an alternative technique called single-nucleus RNA sequencing (snRNA-seq) has emerged, replacing whole-cell sequencing with nuclear sequencing to circumvent this stringent requirement (45, 46).

Subsequently, the recognition of sequencing data at the single-cell level presents another challenge. Scientists have employed micro-reaction systems with special tags/indices, comprising single cells, functional beads, and reverse transcriptomes, to identify cell types and intracellular molecules. In Drop-seq, each functional bead is labeled with four components: a constant sequence, a cell barcode, a unique molecular identifier (UMI), and an oligo-dT sequence from 5′ to 3′. Specifically, the cell barcode is distinct in each bead to label single-cell, while each single-cell has its own UMI to distinguish polymerase chain reaction (PCR) duplicates. Simultaneously, the constant sequence serves as a consistent priming site for future PCR and sequencing, and the oligo-dT sequence facilitates the acquisition of mRNA (39). Moreover, the UMI can mark other types of molecules, such as genes and proteins, enabling multi-omics analysis (47). These ingenious designs ensure the accurate recognition of molecular characteristics at the single-cell level. Following the labeling of each cell’s molecules with a barcode, *in vitro* transcription, PCR, and next-generation sequencing are performed.

**Figure 2:**
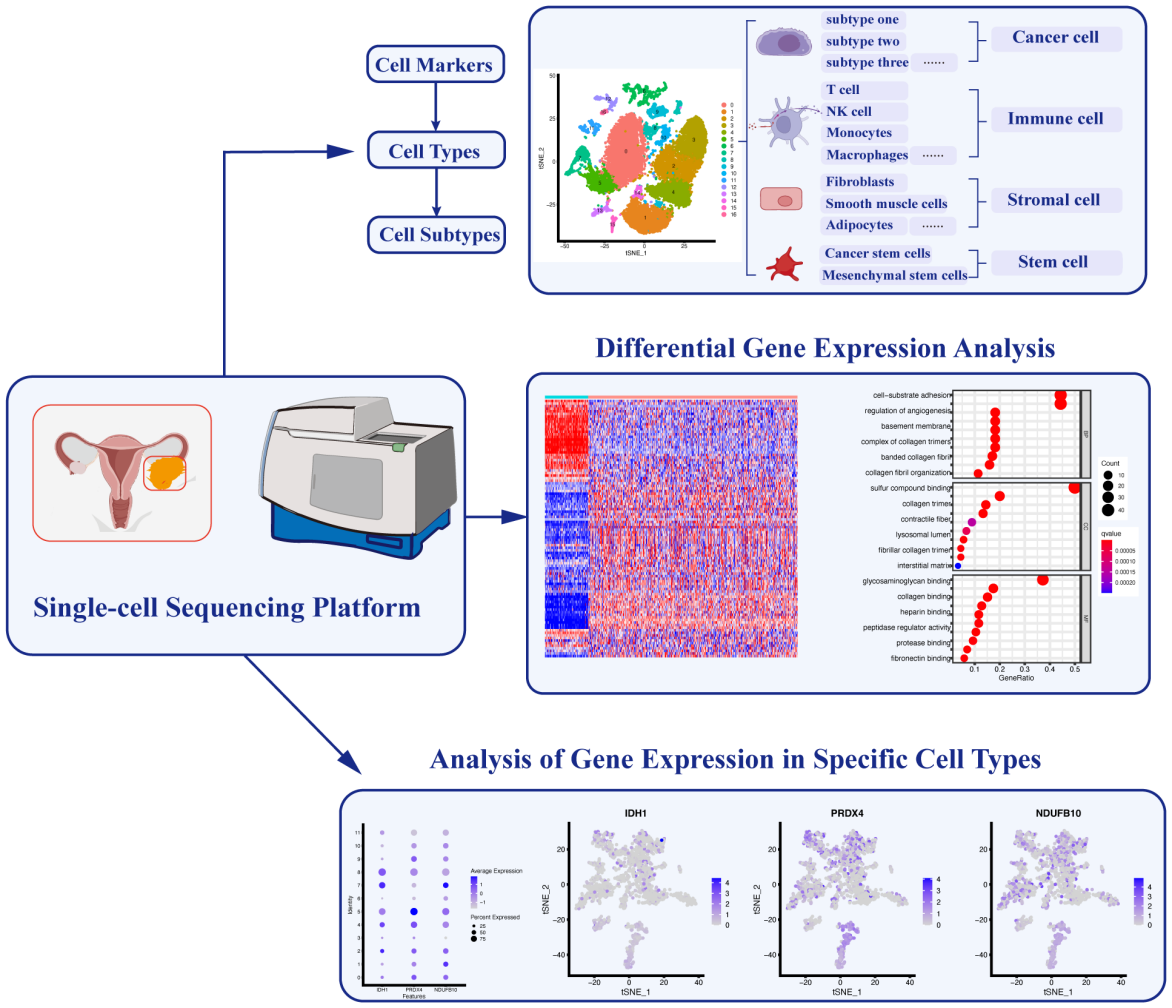
Applications of Single-cell Sequencing Technology in Ovarian Cancer. Single-cell sequencing technology enables the identification of specific cell markers, facilitating the classification and characterization of diverse cell types and subtypes. Furthermore, analyzing differential gene expression among these distinct cell populations allows for a comprehensive understanding of their functional roles. Additionally, the identification of key gene expression signatures in different cell types helps elucidate specific cellular functions within the complex ovarian cancer microenvironment.

Finally, sequencing data analysis is crucial for the significant findings in single-cell sequencing research (48). Multiple specialized programs based on professional bioinformatic methods are used for the analysis of sequencing data (49, 50). The fundamental data analysis process consists of various steps: data acquisition, data cleaning and normalization, gene identification and cell type assignment, cell trajectory analysis, and cell-gene associations. Firstly, the original nucleotide sequencing reads are initially mapped to the transcriptome and read counts and UMIs are utilized to calculate gene expression in individual cells. Subsequently, the data cleaning encompasses the removal of low-quality genetic information and cells with low quality, such as doublets or empty droplets, and high percentages of mitochondrial expression. Following this, data normalization is carried out to standardize gene expression profiles across cells. Next, an essential step is cell type identification, involving the clustering of cells based on their expression patterns, often employing differential expression analysis and known cell markers for validation. Subpopulation analysis further refines cell types, identifying distinct subgroups within them. Then, cell trajectory analysis, which includes pseudotime analysis, reveals the developmental paths and fates of individual cells. Finally, the exploration of cell-gene associations unveils gene modules and regulatory networks, providing insights into functional pathways (**Figure 2**). This multi-step process ensures the scientific integrity and reliability of the findings.

The effective application of Single-cell sequencing relying on robust computational tools and software packages. Various versatile tools have emerged, each possessing unique features and capabilities. Seurat represents a widely adopted R package for the analysis of single-cell RNA-seq data (51, 52). It provides comprehensive workflows encompassing quality control, normalization, dimensionality reduction, clustering, and visualization. In addition, Scanpy, a potent Python library, distinguishes itself with exceptional scalability and speed, making it ideal for the analysis of extensive single-cell datasets (53). Notably, scVI has emerged as a probabilistic framework designed for the integration of scRNA-seq data and the imputation of missing values, a valuable asset when handling noisy or sparse datasets (54). These tools assume pivotal roles in unraveling the intricate landscapes of single-cell data, thereby providing invaluable insights into cell types, developmental trajectories, and regulatory networks.

In summary, single-cell sequencing is a breakthrough technology compared to bulk sequencing, which offers several significant advantages in cancer research: the ability to detect tumor heterogeneity, the ability to discover new cancer cell subtypes, the ability to create a precise map of the TME and analyze immune-related pathological processes, and reveal the molecular mechanisms underlying tumor progression (55). The new discoveries brought about by single-cell sequencing have greatly enhanced our understanding of various diseases and provided novel insights into therapy and diagnosis. Several reviews have summarized the applications of single-cell sequencing in various disorders, such as heart (56) and brain disease (57). However, limited articles have reviewed the advancements of this novel technique in cancer of ovary. Consequently, we summarized the latest research on OC up until March 2023. Subsequent chapters will present detailed single-cell sequencing-based studies of OC to provide precise predictions and guidance for the future development of this field.

## 4 Identification of heterogeneity and cancer cell subtypes in OC

**Figure 3:**
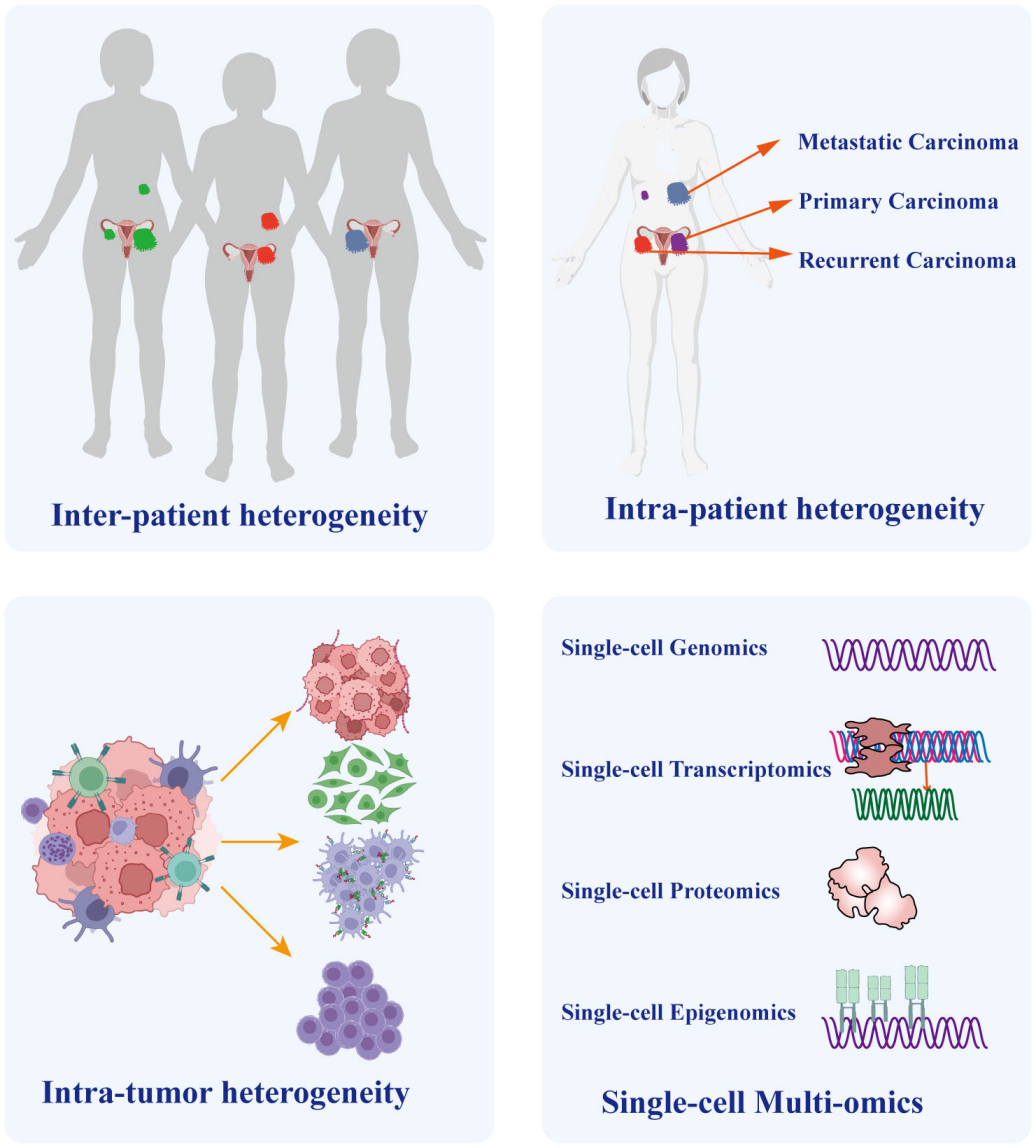
The Crucial Role of Single-cell Sequencing in Exploring Ovarian Cancer Heterogeneity. Heterogeneity Single-cell sequencing technology plays a vital role in unraveling the heterogeneity of ovarian cancer. It enables precise discrimination of tumor heterogeneity among different patients and distinguishes heterogeneity between distinct lesions within the same patient, such as primary and metastatic lesions. Importantly, given the diverse cellular composition within tumor tissues, single-cell sequencing also identifies cellular heterogeneity within the tumor microenvironment. Furthermore, the integration of multi-omicss data, including genomics, transcriptomics, proteomics, and epigenomics, provides a comprehensive understanding of tumor molecular characteristics and underlying developmental mechanisms.

Heterogeneity arises from a combination of genetic and non-genetic factors (58, 59). Cancer cells undergo a progression from normal cells, accumulating genetic and epigenetic mutations that lead to the development of distinct lineages and specialized subtypes. Consequently, despite originating from the same tumor, these cells exhibit diverse mutation types and phenotypes (60, 61). Different cancer cell types exhibit remarkable variability in almost every phenotype, including the potential to form metastasis and develop resistance to chemotherapy (58). The aforementioned reasons account for the extensive heterogeneity in cancer, especially in OC. Intra-tumor heterogeneity plays a crucial role in chemotherapy resistance and even treatment failure in OC (62). Meanwhile, inter-tumor heterogeneity is crucial for individualized treatment (63). It encompasses variations in molecular characteristics of the same type of cancer across different patients, primarily characterized by diverse gene mutations, distinct cell populations, and varying levels of immune infiltration. Therefore, it significantly impacts the outcomes of identical therapeutic strategies on different patients (64, 65). To address this situation, reliable prediction of drug targets provides the basis for personalized cancer treatment. Previous research on target speculation primarily focused on identifying bulk tumor tissues consisting of a variety of immune cells, fibroblasts, endothelial cells, and cancer cells. However, conventional sequencing approaches applied to bulk samples face challenges as they can be complicated by the presence of numerous other cells within the same lesion, which obscures the phenotypes of small cell subtypes. Consequently, traditional bulk sequencing techniques that pool cells together exhibit limited ability to accurately depict prognostic and therapeutic target genes. Moreover, treatment strategies based on next-generation sequencing exhibit diverse treatment responses, suggesting the simultaneous emergence of different cancer cell subtypes within individual patients, as demonstrated by single-cell analysis (66). Single-cell sequencing provides a solution to overcome this challenge by identifying and characterizing cell subpopulations, thereby elucidating tumor heterogeneity (**Figure 3**).

In 2011, a bulk RNA-seq-based study revealed that HGSOC encompasses four molecular types: differentiated, proliferative, mesenchymal, and immunoreactive (17). However, single-cell sequencing analysis detected the coexistence of all four molecular types within a single HGSOC sample (67). Accordingly, to obtain the prognostic value and guide therapeutic decisions, the identification of molecular subtypes should be performed at the single-cell level, given that different molecular types exhibit diverse responses to treatment (68). Over the years, several studies have employed single-cell sequencing to investigate genetic diversity in OC. In 2017, a pioneering study utilized scRNA-seq to explore the genetic profiles of 66 single cells derived from an HGSOC patient (67). The analysis revealed two major cell types: epithelial cells characterized by the expression of proliferation-related genes, and stromal cells marked by elevated levels of extracellular matrix (ECM) and epithelial-mesenchymal transition (EMT) related genes. Although this study provided preliminary insights into the single-cell transcriptome landscape of OC, it was limited in its ability to comprehensively analyze intratumor heterogeneity and identify an adequate number of cancer cell populations. Subsequently, another research group conducted high-throughput scRNA-seq analysis on both primary and metastatic carcinoma lesions (69). Their findings revealed that while primary tumors exhibited significant heterogeneity among individual patients, the gene expressions in metastatic tumors were remarkably homogeneous, but distinct from those in primary tumors (69). The study identified 16 cell types, including four major subtypes of malignant epithelial cells, as well as distinct cell populations associated with high-grade cancer, low-grade cancer, and benign tumors. Furthermore, the composition of cell types underwent significant changes during the transition from primarily epithelial cells in the primary lesions to immunologic cells in the metastatic lesions. Notably, the key cell populations responsible for secreting soluble factors also transformed from myeloid cells to fibroblasts, which might have originated from the epithelial cells. These findings highlight the potential of single-cell sequencing in unravelling the mysteries of tumor and cellular heterogeneity, leading to a better understanding of OC progression.

In a recent study, researchers investigated the intra-tumor heterogeneity of OC using scRNA-seq and identified six cell types: epithelial cells, fibroblast cells, T cells, B cells, macrophages, and endothelial cells (70). Notably, the subtypes of epithelial cells exhibited novel molecular markers for HGSOC, while the subtypes of fibroblast cells revealed heterogeneity in their biological functions. Moreover, the analysis of the genetic regulatory network and the interaction between epithelial and fibroblast cells revealed the potential involvement of the JUN pathway in OC, highlighting it as a prospective therapeutic target. The concept that genetic heterogeneity contributes to the persistence of the majority of cancer cells has been substantiated (71). Moreover, the diverse phenotypic heterogeneity (non-genetic heterogeneity) arising from a similar gene background contributes to crucial malignant characteristics of cancer, such as metastasis and drug resistance. Gene heterogeneity is often acquired, whereas phenotype heterogeneity typically arises from the original cells due to various mechanisms, including epigenetic regulation (72, 73). Identification of the origin-cell state may become novel approach to elucidate the phenotype heterogeneity of a cancer subtype (72, 74, 75). Therefore, single-cell sequencing has been utilized to investigate the link between subgroups of normal FTE cells and HGSOC cell subgroups (10). Six subpopulations of FTE cells, including four secretory subtypes, were identified, and a gene characteristic that can predict a poor prognosis for highly EMT OC was documented. Furthermore, these results revealed a strong association between secretory FTE cells and HGSOC subtypes, highlighting the phenotypic heterogeneity of cancer cells originating from the original cells. This study introduced a novel approach to accurately predict cancer biological processes by assessing original non-malignant cells. This innovative research has shifted the focus of investigating tumor heterogeneity by examining the connection between cancer cells and normal FTE cells, which are believed to be the source of ovarian cancer. Another study using scRNA-seq identified two subclasses of cancer cells associated with poor prognosis in HGSOC (76). Specifically, one group consisted of primary and stem-like tumor cells, and SLC3A1 and PEG10 served as genetic indicators for tumor-initiating cells. The other group was characterized by the presence of CA125 and a decreased quantity of infiltrating cytotoxic T cells. Cell interaction with these malignant cells was mediated by LGALS9 and GAS6, inhibiting cytotoxic T cells. Moreover, these two cancer cell groups can survive during initial therapy and induce immunosuppression. These results suggest a novel indicator for diagnosing and treating OC by targeting the malignant cell subclass.

Subsequently, a multi-omics study investigated the progression timeline of HGSOC subtypes by performing an integrated analysis of total copy number variations and gene expression using single-cell technology (77). This study indicated that the differences in subtypes occur during the late stage of cancer progression. Additionally, recurrent OC was characterized as a proliferative cancer, characterized by gene variability and lack of immune infiltration. In contrast, well-differentiated tumors were characterized by gene stability and high levels of immune infiltration. Moreover, significant heterogeneity gives rise to various subclasses of HGSOC, but a non-constant subclass exists among malignant epithelial cells. This research highlights the need to update previous subtype analyses based on bulk transcriptome sequencing with more stable single-cell multi-omics analysis. Similarly, another research team employed scRNA-seq to analyze the heterogeneity of OC, normal ovary, and embryo samples (78). Eight distinct cell types were identified, and genetic expression analysis revealed similarities between embryo and OC samples in several cell types. A subgroup of malignant epithelial cells derived from embryos was characterized by the presence of PEG10+ during the intermediate phase of progression from embryo to cancer and was associated with carcinogenesis and poor prognosis. Furthermore, the presence of PEG10+ is a significant characteristic of cancer stem cells, given that it influences their self-duplication and promotes resistance to cisplatin through the NOTCH signaling pathway. Therefore, the novel insights into the evolution from embryo to OC can enhance our understanding of carcinogenesis and facilitate the discovery of new therapeutic targets, such as PEG10+.

It is now understood that the tumor-stromal component significantly affects the accuracy of prognostic prediction based on molecular subclasses; however, a comprehensive understanding of stromal cell characterization in OC and their role in classifying pathological subtypes is still lacking. To address this knowledge gap, a study utilized scRNA-seq to identify phenotypes of stromal cells and discover novel potential therapeutic targets in HGSOC (79). The findings revealed that myofibroblasts, fibroblasts, mesothelial cells, and lymphatic endothelial cells are associated with poor survival, while plasma cells are linked to a favorable prognosis. Importantly, different molecular subgroups exhibit distinct stromal cell phenotypes; for example, the mesenchymal type displays a fibroblast phenotype, while the immunoreactive type exhibits an immune cell phenotype. Moreover, a phenotype indicative of poor prognosis is correlated with an unfavorable outcome of the corresponding molecular subtype. The diverse phenotypes resulting from stromal admixture provide insights into the association between molecular subgroups and prognosis and highlight the limitations of previous molecular typing in predicting poor prognosis. In the future, classifying patients based on phenotype biomarkers may serve as a viable approach for precise prognosis and targeted therapeutic interventions.

The wide utilization of single-cell sequencing technology enables the generation of more comprehensive information about OC, including its heterogeneity, molecular subtypes, and diverse cell populations (**Table 2**). These findings contribute to the discovery of prognostic biomarkers and the advancement of targeted therapies. Encouragingly, immunotherapy and targeted therapy, such as PD1/PD-L1 inhibitors and PARPi, based on the molecular phenotype, have been recommended as first-line treatment in combination with carboplatin and paclitaxel (80).

**Table 2:** Key findings related to tumor heterogeneity and identification of cancer cell subtypes obtained using single-cell sequencing.

Tumor	Technology	Key findings	References
HGSOC	scRNA-seq	Coexistence of all four molecular types (differentiated, proliferative, mesenchymal, and immunoreactive) within a single HGSOC sample	(62)
Serous OC	scRNA-seq	Primary tumors showed significant heterogeneity among individual patients, whereas gene expressions in metastatic tumors were remarkably homogeneous, but distinct from those in primary tumors	(64)
HGSOC	scRNA-seq	Identified six cell types within ovarian cancer, including epithelial cells, fibroblast cells, T cells, B cells, macrophages, and endothelial cells, and the JUN pathway is a promising therapeutic target.	(65)
HGSOC	scRNA-seq	Revealed a strong association between secretory FTE cells and HGSOC subtypes, highlighting the phenotypic heterogeneity of cancer cells originating from the original cells.	(9)
HGSOC	scRNA-seq	Identified two subclasses of cancer cells associated with poor prognosis in HGSOC, which can survive during initial therapy and induce immunosuppression	(71)
HGSOC	Multi-omics Analysis of single-cell- seq	Significant heterogeneity gives rise to various subclasses of HGSOC, but a non-constant subclass exists among malignant epithelial cells.	(72)
OC	scRNA-seq	Analyze the heterogeneity of OC, normal ovary, and embryo samples; Eight distinct cell types were identified, and genetic expression analysis revealed similarities between embryo and OC samples	(73)
HGSOC	scRNA-seq	Myofibroblasts, fibroblasts, mesothelial cells, and lymphatic endothelial cells are associated with poor survival, while plasma cells are linked to a favorable prognosis.	(74)

HGSOC, high-grade serous ovarian cancer; scRNA-seq, single-cell RNA sequencing; FTE, fallopian tube epithelium; OC, ovarian cancer.

## 5 Exploration of immune-related signatures and comprehensive landscape of TME in OC

**Figure 4:**
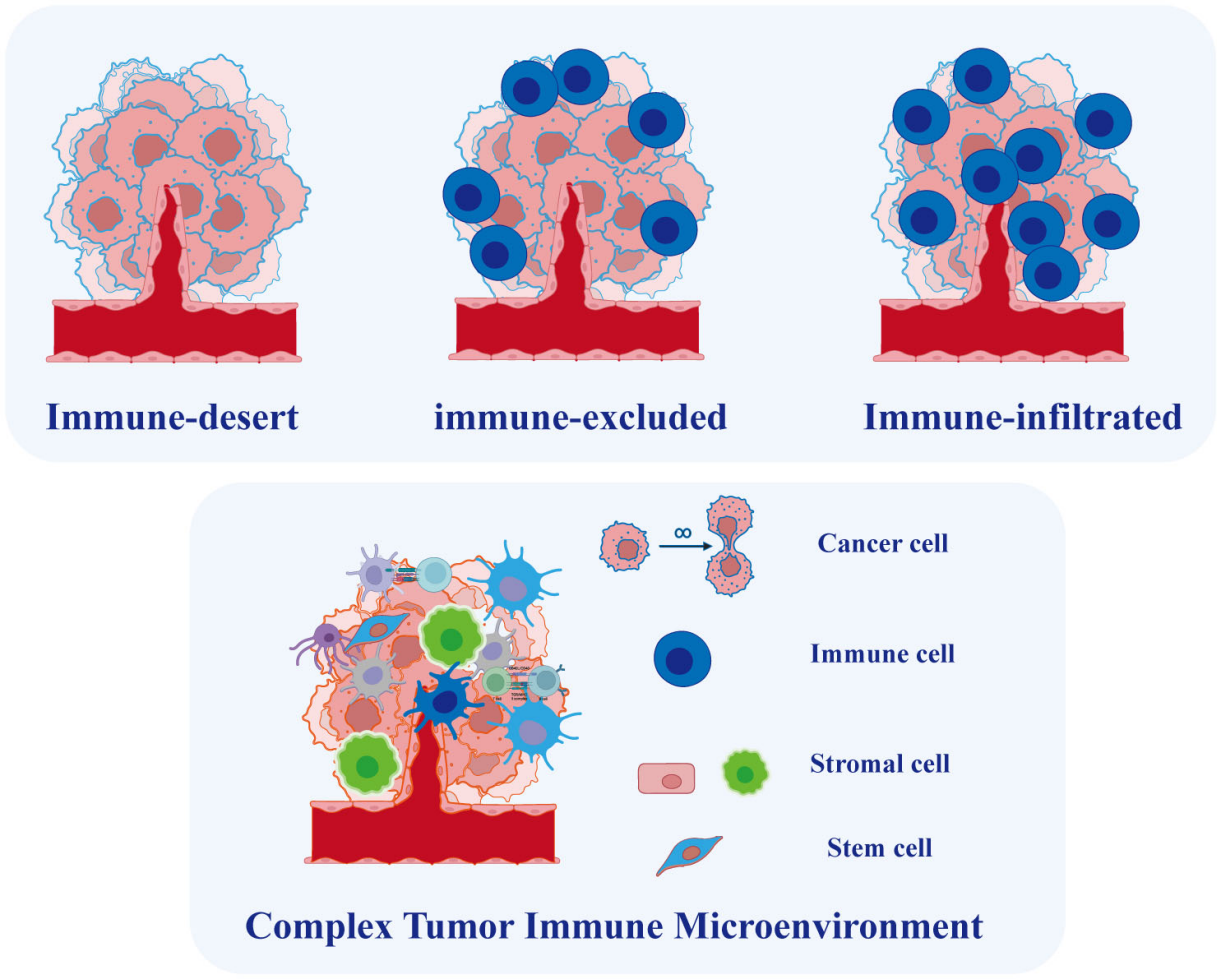
Tumor Microenvironment in Ovarian Cancer. Based on the degree of immune cell infiltration, ovarian cancer can be categorized into immune-infiltrated, immune-excluded, and immune-desert types. The tumor microenvironment in ovarian cancer is complex, comprising various cell types whose interactions play a crucial role in tumor initiation and progression.

OC is widely recognized as an immunogenic tumor and the TME in OC is closely linked to immunotherapy efficacy and prognosis (81). The TME is a complex ecosystem comprising malignant cells, immune-infiltrated cells, stromal cells, and non-cellular elements (**Figure 4**). Various types of immune cells infiltrate the TME, such as clusters of myeloid-derived suppressor cells, lymphocytes, macrophages, mast cells, neutrophils, and dendritic cells, playing crucial roles in tumor progression, recurrence, and metastasis, with both favorable and unfavorable effects (82). The heterogeneous TME in ovarian cancer OC mirrors the intertumor diversity of this malignancy. For instance, a study identified varying immunological molecular characteristics across different degrees of metastatic lesions in a patient with HGSOC who underwent multiple chemotherapies (83). Furthermore, the coexistence of inflammatory and non-immune infiltrated microenvironments was observed in various untreated patients with HGSOC, suggesting significant variation in infiltrating immune cells (84). The inherent heterogeneity of the TME in OC makes it challenging to conduct effective therapies (81). Moreover, tumor metastasis in OC has been strongly linked to the TME (85). Malignant cells in EOC have a higher propensity for metastasis and the establishment of a peritoneal microenvironment compared to solid tumors (86). Consequently, investigating TME heterogeneity is imperative for identifying new therapeutic targets and prognostic markers. While the molecular classification of OC is based on traditional sequencing, single-cell data plays a crucial role in advancing personalized immunotherapy.

Over the years, single-cell sequencing has been extensively employed in various studies to identify novel immunotherapy approaches for diverse cancers (31, 87). This technology offers significant advantages in exploring tumor immune infiltration, immune cell types, and the interaction between immune cells and malignant cells (88). Encouragingly, numerous novel therapies targeting anti-angiogenesis, anti-fibrogenesis, and immune checkpoint inhibition have emerged to combat the TME (89). While various immunotherapies prove beneficial in the presence of specific biomarkers, their effectiveness varies among patients due to the heterogeneity of the TME. For instance, checkpoint inhibitors aimed at restoring CD8+ T cell function demonstrated unsatisfactory responses in OC patients (90). Therefore, conducting a comprehensive analysis of immune heterogeneity and the composition of tumor tissue is highly advantageous in identifying potential immunotherapeutic targets and predicting prognosis based on the immune status. The TME in ascites also plays a crucial role in tumor development. Ascites is characterized by multiple cell types and a complex microenvironment (91). To comprehensively characterize the TME of ovarian carcinoma ascites, a study employed single-cell sequencing technology to analyze ascites samples from 22 OC cases (9). Like primary ovarian tumors, ascites exhibits significant inter-patient heterogeneity, primarily in distinct cancer-associated fibroblast (CAF) populations and immune cell subtypes. Interestingly, the JAK/STAT pathway was activated in both CAFs and malignant cells, suggesting its potential as a crucial mechanism driving ascites development and drug resistance in OC. Currently, a clinical trial (NCT02713386) is investigating the efficacy of ruxolitinib, a JAK/STAT pathway inhibitor, in treating of HGSOC (92).

To capture both the spatial distribution of immune infiltrates and their overall expression, researchers proposed the concept of the immunity continuum (93, 94). This novel concept defines three immunophenotypes based on the spatial distribution and extent of T cell infiltration: immune-infiltrated (T cells infiltrate the tumor epithelium), immune-excluded (T cells accumulate in the stromal region), and immune-desert phenotypes (low or no T cell infiltration) (**Figure 4**). The distinct infiltration patterns lead to inconsistent responses of OC to immunotherapy. Hornburg et al. recently employed single-cell sequencing to investigate the composition of the OC immune microenvironment and identify the immunophenotypes (95). They identified distinct features in the immune-desert phenotype of OC, including enhanced metabolic pathways, reduced antigen presentation, and an enrichment of monocytes and immature macrophages. Furthermore, the infiltrated and excluded phenotypes of cancer exhibit differences in immunocyte and fibroblast subpopulations. Specifically, the immune-excluded phenotype showed an enrichment of pre-dysfunctional CD8+ GZMK T cells, while the immune-desert phenotype exhibited enrichments of FCN1 monocytes and MARCO macrophages. Multiple cytochemokine receptor-ligand interactions were identified in the TME, including CXCL16-CXCR6-mediated communication between tumor cells and immune cells and CXCL12/14-CXCR4-mediated communication between stromal cells and immune cells, potentially regulating the phenotype of immune infiltration (95). While immune cells infiltrating metastatic lesions do not consistently exert positive effects, T cell always plays a central role in immunotherapy methods (96). Another study employed single-cell approach to reveal diverse immunological cell clusters and subtypes, elucidating their potential roles in anti-tumor immunity and the progression of OC metastasis (97). The researchers categorized these OC cases into two groups based on T cell infiltration: the high-infiltrated T cell group and the low-infiltrated T cell group. The results revealed an enrichment of TOX+ CD8+ and granulysin+ CD4+ T cell subtypes in the high-infiltrated T cell group. Additionally, a distinct population of MKI67+ plasmablasts was identified within this group. Lastly, NR1H2+ IRF8+ and CD274+ macrophage subtypes were discovered, demonstrating their positive response to tumor cells. These newly identified immune cell subtypes with cytotoxic properties during the anti-tumor immune response may provide potential targets for treating of ovarian tumors.

While HGSOC with immune infiltration generally exhibits a more favorable prognosis than other types (98), it can still exhibit resistance to immunotherapies (99). Therefore, investigating different types of tumor-infiltrating lymphocytes is significant due to this discrepancy. A research team identified a triple-positive marker, comprising the co-expression of CD39, CD103, and PD-1, for tumor-infiltrating lymphocytes in HGSOC (100). Specifically, the triple-positive CD8+ T cells exhibited reduced receptor diversity and high gene expressions associated with cytolysis and humoral immune response. Meanwhile, triple-positive Tregs displayed receptor diversity and tumor residency. The triple-positive tumor-infiltrating lymphocytes exhibit remarkable prognostic capabilities and suggest potential combination targets for immunotherapies, including CD39, PD-1, and TIGIT.

The EMT determines the characteristics of ovarian tumors (17, 101), and it plays a crucial role in tumor metastasis, invasiveness, and drug resistance, and indicates poor survival in HGSOC patients (102, 103). Therefore, it is imperative to further explore detailed information about EMT in the TME to advance innovative immunotherapy strategies. Subsequently, a study depicted the ecosystem landscape of early- and late-stage HGSOC by analyzing the heterogeneity of the TME and the characteristics of EMT using single-cell sequencing (104). The notable findings include a prognostic prediction model comprising four EMT-related genes (NOTCH1, SNAI2, TGFBR1, and WNT11) associated with poor survival. Additionally, the dominant cancer-associated fibroblasts were characterized by the presence of five distinct genes (*α*-SMA, vimentin, COL3A, COL10A, and MMP11), which contribute to the development of EMT features and enhance tumor invasiveness. Moreover, the study suggested that TIGIT blockade could serve as a potential therapeutic strategy in HGSOC.

Immunotherapies in OC have shown limited efficacy, primarily due to an incomplete understanding of their biological potencies and underlying mechanisms. For example, immune checkpoint inhibitors have demonstrated significant efficacy in various solid tumors; however, their effectiveness in OC is comparatively lower (105, 106). Relevant studies have indicated that the functional capacity of T and NK cells, rather than their abundance, is critical in the context of immunotherapy (107, 108). Therefore, it is crucial to investigate the response of diverse cell types, especially immune-related cells, to immunotherapy drugs and understand the associated alterations in their cellular states. A research team performed immune function and scRNA-seq analyses using an innovative co-culture model of HGSOC organoids and immune cells treated with a combination of anti-PD-1 and anti-PD-L1 antibodies, aiming to compare the effects with those of traditional anti-PD-1 or anti-PD-L1 monotherapies (109). The novel antibody induced favorable alterations in immune-associated cells, particularly leading to a shift in NK cells and a subtype of CD8+ T cells into an aggressive and cytotoxic state. These findings demonstrate that state changes in immune cells are crucial for the effectiveness of immunotherapy, and bispecific antibodies can elicit a superior immune response compared to monospecific antibodies. In conclusion, OC has huge potential as a viable target for immunotherapies, provided that these treatments employ precise mechanistic approaches targeting specific immune-related cell clusters. Then, a study conducted single-cell and bulk RNA sequencing of blood samples from patients with EOC after neoadjuvant chemotherapy (NACT) to examine the function of circulating immune cells in the periphery (110). Single-cell examinations revealed an increased abundance of central memory CD8+ T cells and regulatory T cells during NACT, as well as an upregulation in the expression of monocyte and HLA class II genes. Thus, chemotherapy alleviated immune suppression by enhancing antigen presentation. Furthermore, the levels of CD8+ T cells and the expression of PD-1/PD-L1 in circulating T cells in the peripheral circulation did not increase. These promising findings have important implications for the efficacy of immune checkpoint inhibitor drugs in the treatment of OC.

In conclusion, these novel single-cell sequencing technologies provide insights into the functional and spatial characteristics of the tumor immune microenvironment, highlighting the importance of personalized immunotherapies to address the heterogeneity of the TME. By conducting single-cell sequencing, comprehensive information about immune responses, which was previously obscured in bulk sequencing, can be elucidated, leading to the discovery and application of new therapeutic targets and innovative methods.

## 6 Investigation of the drug resistance mechanisms in OC

The clinical progression of advanced ovarian malignancy is characterized by its aggressiveness. Initially, it exhibits a favorable response to platinum-based chemotherapy, but the majority of patients experience relapse over time, ultimately developing resistance to platinum-based medications (111). Despite the fact that initial treatment is effective in approximately 70% of patients with HGSOC (112), chemoresistance often emerges during the course of therapy, posing a significant obstacle to successful treatment (113). The improvement of current therapeutic strategies for OC strongly depends on our understanding of the molecular mechanisms underlying drug resistance and the identification of specific cell subtypes with inherent resistance to chemotherapy. However, it remains unknown whether resistant clones arise due to genomic instability or therapy-related genome damage during tumor formation or as a result of chemotherapy. A study demonstrated that drug resistance observed in clinical practice follows a continuum, resulting from Darwinian evolutionary selection of inherently resistant clusters among diverse subtypes of cancer cells (114). Identifying these subgroups with unfavorable characteristics, such as tumor stem cells or clusters exhibiting chemoresistance, may unveil the molecular mechanisms activated within these subgroups. These findings will be valuable for clinical practice when accessible therapies targeting these molecular “switches” are developed. Single-cell techniques enable comprehensive interpretation of the transcriptional and genetic changes associated with chemotherapy in individual malignant cells. Furthermore, it allows for a detailed examination of the interaction between tumor cells and their microenvironment.

To investigate the mechanisms underlying chemoresistance in HGSOC, a research group prospectively collected tumor specimens before and after chemotherapy and conducted single-cell transcriptome analyses with high-resolution (115). They observed an increasing trend in stress-related cell states during chemotherapy by removing confounding patient factors using an innovative analytical method called PRIMUS. This observation was further validated through *in-situ* RNA hybridization and bulk sequencing. Moreover, stress-related conditions were initially present in the tumor tissue, and the associated subclones consistently expanded during chemotherapy, ultimately resulting in unfavorable survival outcomes. The presence of inflammatory cancer-associated fibroblast subgroups within tumors suggests a drug-induced stress response in both malignant and stromal cells, triggering a paracrine feed-forward loop (115). Notably, tumor stroma has been recognized to play a crucial role in drug resistance across various malignancies, and an increased stromal component in OC is associated with chemotherapy resistance (116, 117). Finally, they identified a specific condition of chemoresistance that involves both stromal signaling and subclone development at the single-cell level.

A different research team investigated the single-cell properties associated with the gradual development of carboplatin resistance in OC (118). In this study, multiple isogenic clones attained similar levels of drug resistance following successive treatment cycles; however, there was considerable heterogeneity in their transcriptomes. Two traits have been linked to chemoresistance: extensive inhibition of the P phase found in all clones in the initial phases of resistance development and recurring activation of the Q phase detected in a particular clone in the later stages of treatment. Furthermore, distinct clones associated with resistance were found to induce an IFN response during the P phase or activate Wnt signaling during the Q phase. These findings suggest a theory of hysteresis in resistance, specifically the transition between the P and Q phases, which influences the dynamics of drug exposure and resistance. Additionally, they established a novel method for monitoring cellular states during chemotherapy to enhance efficacy. A separate study examined the development of resistance in HGSOC cells following chemotherapy (119). Analysis of single-cell RNA sequencing data obtained from HGSOC tumors collected during therapy revealed three distinct phenotypic archetypes that contribute to the progression of HGSOC. These archetypes, which exhibit characteristics related to metabolism and proliferation, cellular defense response, and DNA repair signaling, were consistently observed among all patients. The analysis uncovered a notable transition towards the metabolism and proliferation archetype in cancer cells subjected to multiple lines of treatment, suggesting the emergence of specialized subclones within the tumor.

Over the years, numerous studies have demonstrated that diverse drug responses can be attributed to the co-progression of cancer and non-cancer cells (83, 120). The increased infiltration of CAFs may contribute to the reduced response rates observed with certain therapies, including immune checkpoint inhibitors, as their efficacy is influenced by the TME (121). This theory was validated through a single-cell experiment, demonstrating the interaction between CAFs and macrophages in modulating the biological processes of OC cells within the ascites microenvironment (9). Specifically, CAFs activate the JAK/STAT pathway in malignant cells by secreting IL-6, leading to reduced survival rates and the emergence of chemoresistance (9).

In summary, drug resistance may not be linked to gene mutations but instead be associated with specific resistant subclones or cellular phenotypes, which reflects the heterogeneity of tumors. Studies conducted in other solid tumors have also shown a close relationship between the emergence of drug resistance and cancer heterogeneity (122). However, the intra-tumor heterogeneity of malignant cells in OC, as well as the heterogeneity of related non-malignant cells, remains poorly understood despite their significant roles in driving chemoresistance. Therefore, research into the mechanisms of therapeutic resistance in OC is still in its early stages. Our current understanding only begins to uncover the fundamental mechanisms responsible for drug resistance, with many aspects still awaiting complete clarification. Encouragingly, single-cell sequencing provides support for researchers to describe the mechanisms of chemoresistance in OC at a high-resolution level.

## 7 Investigation of metastatic dissemination in OC

Over 90% of tumor-related deaths are attributed to the intricate biological process of cancer metastatic dissemination (123). Various metastatic patterns and mechanisms have been proposed during tumor progression, including lymphatic metastasis, hematogenous spread, and intraperitoneal implantation of malignant cells (124); however, they remain poorly understood. During tumor metastasis, metastatic cells invade the circulation, survive as CTCs, and subsequently extravasate and seed into distant locations (123). Specifically, OC colonizes the peritoneal cavity by disseminating tumor cells (DTCs) through attachment and seeding (124). The DTC clusters that escape from primary tumors are commonly referred to as the metastatic “seeds” (125). The persistence of DTCs indicates the presence of a subpopulation of non- or slow-cycling tumor cells in primary tumors, which may possess innate drug resistance features or acquire resistance abilities during chemotherapy (126). However, knowledge about metastatic cell subtypes and other mechanisms related to metastasis is still lacking in the current research context, which limits the development of highly effective therapeutics against OC metastasis. Besides tumor cell subpopulations, the TME consists of various stromal cells with distinct molecular characteristics (127). The dissemination of tumor cells is a prerequisite for metastasis and is associated with EMT (128). Within the TME, EMT is primarily regulated by tumor-stroma interactions, particularly through the TGFβ signaling pathway secreted by CAFs (129). Therefore, it is hypothesized that a specific subpopulation may exist in the TME, enabling them to evade chemotherapy and migrate from the primary site, leading to tumor Metastasis. Consequently, the identification of this crucial tumor cell subpopulation in OC may provide novel targets for therapeutic interventions aimed at preventing metastasis and recurrence. The current state of single-cell sequencing technology is highly advanced and well-suited for in-depth exploration of molecular biology at the level of individual cells.

In 2021, a team performed a single-cell transcriptome analysis to uncover the characteristics of HGSOC. Through trajectory analysis, they identified an association between metastatic OC and the dysfunction of various signaling pathways, especially the “translational machinery” pathway (70). Subsequently, a study revealed the multi-omics landscape of HGSOC using single-cell sequencing technology (16). The results demonstrated an identical pattern of genetic expression and DNA methylation in the same genetic lineage of primary and metastatic cancer cells, suggesting that the metastatic cells are likely subclones derived from the original tumor. Next, a team performed scRNA-seq on primary and metastatic tumor tissues of OC (130). They observed up-regulated expressions of ERBB2 and HOXB-AS3 in metastatic samples compared to primary lesions. Furthermore, several signaling pathways were dysregulated in metastatic samples of HGSOC. Additionally, malignant-associated fibroblasts with EMT progression were elevated in metastatic lesions and contributed to angiogenesis and immune regulation. Analysis of clinical outcomes revealed that EMT was associated with a poor prognosis (130). Finally, single-cell sequencing of 6 metastatic omentum lesions from OC unveiled the gene characteristics in the immune microenvironment of metastatic lesions, providing a basis for further investigation into the mechanisms underlying metastasis (97). However, each study has its own specific aims and limitations. A comprehensive solution will eventually emerge as we make incremental advancements in our understanding. As an illustration, one study has revealed a straightforward yet equally significant observation: metastasis necessitates a smaller number of epithelial cells to sustain the microenvironment in comparison to the original tumors (69).

In summary, these findings at the single-cell resolution will provide a comprehensive understanding of metastatic occurrence and progression, leading to the discovery of new therapeutic targets and ultimately improving patient outcomes for OC in the near future. Furthermore, a comprehensive and precise description of tumor heterogeneity, the immune microenvironment, dynamic alterations in cell subtypes, and intercellular interactions in metastatic lesions is crucial for future advanced research.

## 8 Exploration of the initiated recurrences in OC

Following primary debulking surgery and subsequent chemotherapy, 70%-80% of OC patients experience tumor recurrence and gradually develop resistance to first-line chemotherapies (131). Consequently, it is crucial to comprehend the risk factors associated with OC recurrence and develop reliable prognostic models for predicting and preventing recurrence. Intra-tumor heterogeneity and complex tumor biology behaviors are associated with tumor recurrence, drug resistance, and poor survival outcomes (132). In recent years, numerous studies utilizing single-cell approaches have elucidated the principles and characteristics that govern the onset and progression of OC recurrence.

The progress of innovative therapies for OC patients has been hindered by the absence of reliable markers for identifying and targeting relapse initiators. Encouragingly, a research group performed scRNA-seq to identify the “trigger” of recurrence occurrence (26). In this study, pseudotime analysis was utilized to trace the lineage of metastatic malignant cells prior to seeding. Specifically, seven subclusters were identified in the primary tumor, and the CYR61+ “stress” subgroup was deemed as the initiator of recurrence. Additionally, it was demonstrated that a subset of RGS5+ CAFs actively promotes malignant dissemination. Thus, the combination of CYR61+ and RGS5+ was significantly correlated with OC relapse and patient survival. Interestingly, harnessing the high resolution of single-cell sequencing, distinct cellular compositions were observed in untreated and recurrent ovarian tumors (26). In untreated metastatic lesions, immune cells comprised 40% of the total cells, while malignant cells accounted for 30%. In contrast, immune cells constituted only 0.5% of the cell population in recurrent lesions, whereas malignant cells constituted as high as 90%. Furthermore, in untreated malignant lesions, interactions between T cells and apoptotic cancer cells have been observed (26). Conversely, chemotherapy-induced cancer cell dormancy promotes malignancy progression (133), enabling evasion of immune surveillance. Based on the aforementioned findings, it is reasonable to infer that chemotherapy may impact the immune status, resulting in chemotherapy resistance and ultimately leading to cancer recurrence. However, further in-depth investigations are required to validate this inference.

The primary cause of recurrence has been hypothesized to be chemotherapy-induced residual DTCs (127). Furthermore, tumorigenesis, maintenance, and recurrence are also dependent on CSCs, as demonstrated in multiple studies (134, 135). Strong evidence from preclinical models and patient samples indicates that platinum-based chemotherapy promotes the enrichment of drug-resistant aldehyde dehydrogenase positive (ALDH+) ovarian CSCs (136), which play a role in tumor relapse and disease recurrence (137). CSCs are often in a non-active state, rendering chemotherapy targeting rapidly proliferating cells ineffective against them (138, 139). Therefore, tumor recurrence, metastasis, and ultimately, multi-line drug resistance may be strongly associated with CSCs that evade treatment. Notably, the presence of CSCs is of particular interest as a potentially valuable therapeutic target in OC. Although malignant lesions contain a low proportion of CSCs, the identification and targeting of these cells are critical for improving the poor survival rate of ovarian malignancy (134, 135). A study concentrated on markers associated with CSCs in OC and identified stromal cell subtypes that express these markers (67). Their initial analysis using scRNA-seq revealed a small subpopulation of cancer stem-like cells in OC. Similar to previous studies, a team using a single-cell approach was unable to identify cells that co-expressed established stem cell markers (97). However, they did identify a cell population closely resembling embryonic stem cells (ESCs) adjacent to the epithelial cell cluster, which exhibited high expression of the proliferative marker MKI67. The successful identification, isolation, and investigation of putative ESCs may yield valuable insights into the characteristics of CSCs in OC. Further advancement in this field depends on accurately identifying and quantifying the frequencies of CSCs in OC lesions, and single-cell level sequencing represents an effective and accurate method for this purpose.

In conclusion, findings obtained through single-cell analysis provide new insights into OC recurrence and the heterogeneity of cell types in both primary and recurrent lesions. In the future, studies utilizing single-cell technology will elucidate the mechanisms underlying tumor recurrence, leveraging its high-resolution capabilities and multi-omicss analysis.

## 9 Exploration of the origin of OC

Evidence from animal models and genetic studies suggests that HGSOC may originate from the secretory epithelium in the distal fallopian tube (140–142). Furthermore, a study using single-cell sequencing technology provided further confirmation of this theory by revealing the single-cell landscape of normal fallopian tubes (143). Additionally, it was confirmed that PAX8, SOX17, and RUNX3 are potential key factors involved in the differentiation of oviduct epithelial cells, with “secretory” epithelial cells potentially serving as the precursors of most HGSCs. Nevertheless, it remains unclear whether there are distinct subgroups of FTE cells and how these subgroups correlate with OC cell subtypes. In an innovative study, scRNA-seq technology was employed to explore normal fallopian tube tissue and identify molecular subtypes of FTE cells, shedding light on tubal epithelial heterogeneity and its relationship to the origin of serous OC (10). The occurrence of nongenetic heterogeneity in individual tumors is attributed to the unique cell-cell interactions. The researchers identified six molecular subclasses of FTE cells, and their findings demonstrated that investigating the molecular characteristics of FTE cells, which serve as the origin of serous OC, allows for precise quantification of nongenetic heterogeneity in serous OC. The molecularly characterized atlases of benign fallopian tube cells will enhance our understanding of early lesions in OC, potentially facilitating the identification of biomarkers for early diagnosis. Furthermore, another group demonstrated through single-cell transcriptome analysis and developmental trajectory analysis that a tumor cell subtype highly expresses ciliated epithelial markers, confirming that ovarian malignancies originate from FTE cells and retain the characteristics of their progenitor cells (70). Likewise, the genetic signature of certain tumor cell subtypes was described in a recent study, providing further confirmation of their origin from fallopian tube cells (79). These studies overlap in their assertion that single-cell sequencing offers unique advantages in investigating the origin of OC. It allows the tracking of originating cells traits through trajectory analysis and enables accurate analysis of the genetic phenotype similarities between normal cells and malignant cells. Accordingly, exploring the progenitor lineage of malignant cells holds great value for early-stage OC diagnosis by identifying meaningful early diagnostic indicators.

## 10 Spatial transcriptome and multi-omicss analysis based on single-cell sequencing

### 10.1 Spatial transcriptomes

**Figure 5:**
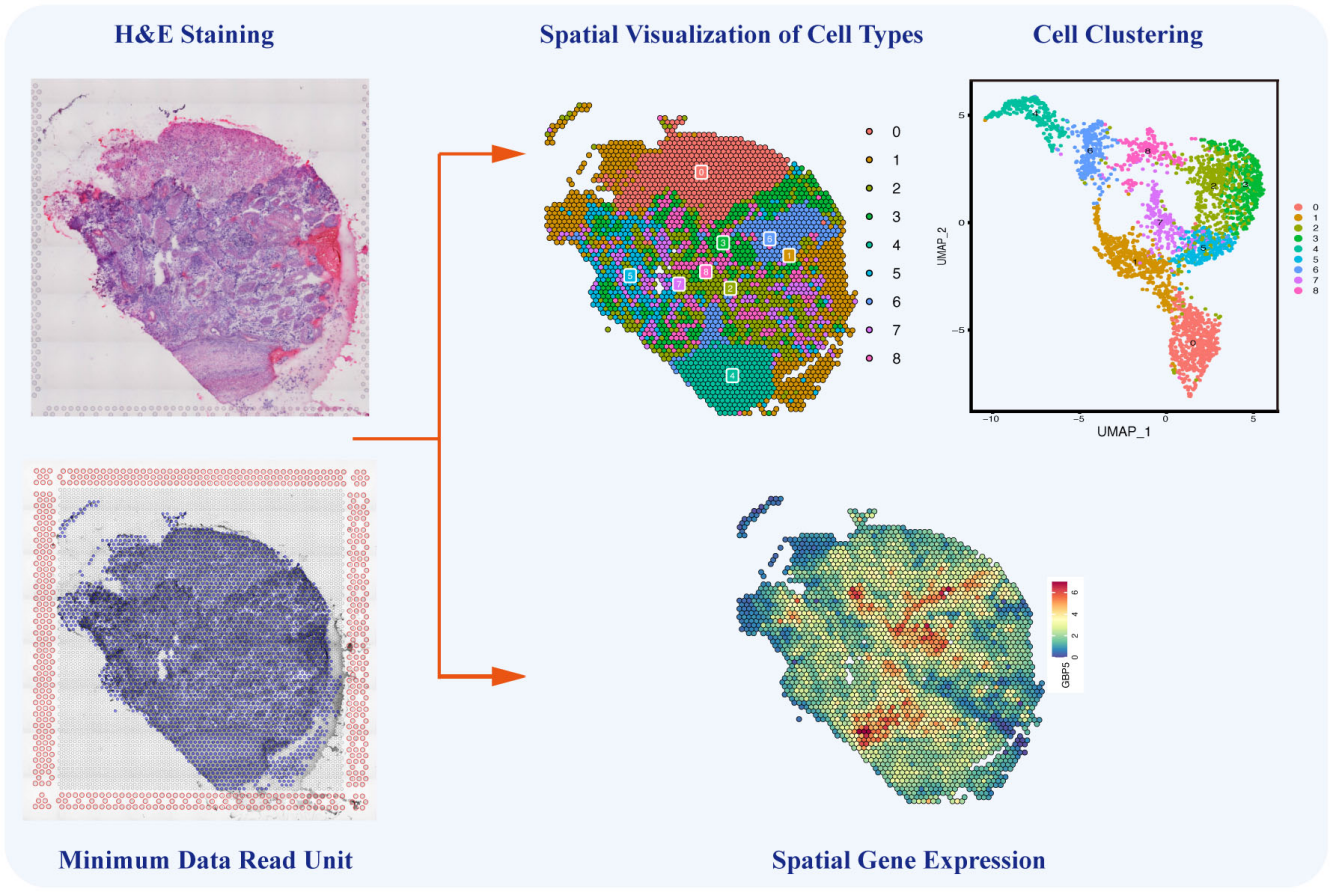
Single-cell Spatial Transcriptomics in Ovarian Cancer. The single-cell spatial transcriptomics technology allows for the spatial reconstruction of cells, mapping different cell types and their interactions onto HE-stained tissue sections. This innovative approach complements the limitations of traditional single-cell sequencing by providing crucial spatial information on cell-to-cell communication and interactions within the ovarian cancer microenvironment.

Single-cell sequencing still has certain technical limitations, including the absence of intercellular spatial information and the challenge of reconstructing the chronological order of cellular evolution. Encouragingly, in 2016, Stahl et al. invented a sequencing method known as spatial transcriptomics, combining sing-cell transcriptomics with spatial positions to decipher cellular composition and intercellular communications in a spatial context (144). By integrating single-cell sequencing with spatial transcriptomics and developing pseudo-time analysis, the above limitations could be overcome, enabling the unrestricted application of single-cell sequencing. As the latest sequencing technology, spatial transcriptomics enables the visualization and quantification of transcriptomes of specific tissue slices in a two-dimensional resolution of specific tissue slices (145). Spatial transcriptomics and temporal lineage tracing stimulate the investigation of the original spatial information, dynamic development, and differentiation of isolated cells. Additionally, spatial information plays a crucial role in understanding cancer heterogeneity and is essential for cancer diagnosis, subtyping, grouping, and therapy (146). This novel technology provides gene expression data from specific regions of tissue sections, enabling the identification of cell types and the analysis of intercellular relationships within these regions (**Figure 5**) (147). Through advanced bioinformatics analyses, the integration of single-cell sequencing and spatial transcriptomics enables the spatial localization of distinct cell subpopulations, annotation of transcriptome location information, and precise analysis of TME heterogeneity from the perspective of intercellular interactions. The powerful integration of these new technologies ensures both the accuracy of tumor-related research and the efficient development of tumor diagnosis methods.

In 2018, 10X Genomics acquired spatial transcriptomics technology and developed the modified sequencing platform known as 10X Visium, which has become the most commonly used platform. In 2020, the first research article utilizing this commercialized platform was published (148). This study integrated single-cell and spatial transcriptome data and identified a tumor-specific keratinocyte in cutaneous squamous cell carcinoma, which plays an important role in intercellular communication. The latest research on OC employed spatial transcriptomics technology to investigate the differential characteristics of HGSOC before and after neoadjuvant therapy (147). This study identified distinct tumor subclones within individual tumor sections, each with unique copy number alteration (CNA) profiles and spatial distributions. Additionally, subclone-specific microenvironments enriched in genes encoding plasma membrane and secreted proteins were uncovered. Similarly, in 2022, a professional team utilized spatial transcriptomics to uncover the mysteries of HGSOC (149). Their study revealed significant spatially defined heterogeneity among patients exhibiting different responses to NACT. Spatial analyses comparing poor and good responders to chemotherapy indicated that spatial interactions among adjacent cell types can influence chemotherapy responsiveness. Moreover, they identified spatially defined cell types within ovarian tumors that exhibited drug resistance, with mesenchymal cells being the most prominent type. Overall, spatial transcriptomics of single-cell shed light on the intra-tumoral subclonal and infiltrative heterogeneity in OC, contributing to a better comprehension of biological characteristic.

The Xenium *in situ* platform, a new spatial transcriptomics product developed by 10X Genomics, offers advanced capabilities for mapping hundreds of transcripts *in situ* at subcellular resolution (150). Building upon the features of the 10X Visium platform, Xenium *in situ* introduces several enhancements, including a larger imageable area and the integration of gene expression data with histological images (including H&E and IF staining) from the same tissue section. The initial Xenium kits support up to 400 gene plexes and allow for the inclusion of up to 100 custom RNA targets that can be added to pre-designed panels. The platform offers a throughput of approximately 2.8 cm^2^ of imageable area per slide, with the potential to reach up to 17 cm^2^ per week. Furthermore, Xenium *in situ* is designed to support even higher throughput, with the capability to handle gene plexes exceeding 1000, as well as enabling concurrent protein and RNA measurements on the same tissue section. A recent study explored eight preliminary Xenium datasets from the mouse brain and two from human breast cancer (151). By comparing scalability, resolution, data quality, capacities, and limitations with eight other spatially resolved transcriptomics technologies, the study found that Xenium represents a significant improvement compared to other RCA-based technologies. Its enhanced detection efficiency and resolution enable the identification of cell types in spatial context, making it a valuable tool for exploring spatial biology. Xenium *In Situ* has already been utilized in breast cancer research to gain biological insights into the progression from ductal carcinoma *in situ* (DCIS) to infiltrating carcinoma and to predict the hormone receptor status of tumor subtypes (150, 152). Researchers identified that PGR may serve as a novel marker for the transition between DCIS and invasive cancer. It is anticipated that Xenium *in situ* will also find applications in ovarian cancer research in the near future.

In 2020, a new spatial transcriptomics technology called GeoMx™ Digital Spatial Profiler (DSP) was developed (153). This technology enables highly multiplexed detection of mRNA targets in formalin-fixed paraffin-embedded (FFPE) tissues. The DSP system employs RNA *in situ* hybridization (ISH) techniques to profile user-defined regions of interest. This is achieved through region-specific cleaving and the collection of photocleaved indexing oligos. The cleaved indices are then quantified using NanoString nCounter^®^ technology, which provides digital quantification of RNA expression with spatial context. The system is user-friendly, minimizes hands-on time, and can deliver highly sensitive digital quantification of protein or RNA expression in less than a 4-day turnaround time. A recent study utilized the GeoMx^®^ Cancer Transcriptome Atlas to correlate the protein profiles of small extracellular vesicles (sEVs) derived from human fallopian tube epithelium (hFTE) with the tissue transcripts of hFTE (154). Spatial transcriptomics analysis revealed cell-type specific transcripts in hFTE that encode sEVs proteins. Notably, proteins such as FLNA, TUBB, JUP, and FLNC exhibited differential expression in secretory cells, which are precursor cells for HGSOC. This study provides valuable insights into understanding the role of hFTE-derived sEVs during the development of ovarian cancer.

### 10.2 Single-cell multi-omics

Single-cell multi-omics sequencings encompass not only transcriptomics but also genomics, proteomics, metabolomics, and epigenetics, providing comprehensive information about OC in a high-throughput manner. For example, single-cell genomics analysis can identify nucleotide variations and polymorphisms (155). Importantly, multi-omics analysis addresses certain limitations of transcriptome analysis alone, such as enabling the distinction of diverse cell subpopulations and the tracing of cellular lineages. The progression of cancer involves not only transcriptome-level variations but also genome mutations, epigenetic regulation, and proteomic changes. Single-cell multi-omicss sequencing can systematically describe tumor heterogeneity and explore molecular mechanisms in multiple dimensions, which can meet the requirements of comprehensive analysis in OC. Therefore, investigating multi-omicss alterations and related epigenetic management provides a landscape of molecular features in OC, which can contribute to improving innovative diagnosis and treatment approaches.

Encouragingly, the field of single-cell multi-omics analysis in OC is rapidly advancing. Recently, a professional team characterized the tumor heterogeneity and elucidated the pathological mechanisms in HGSOC by conducting simultaneous analyses of gene, transcriptome, and epigenome features at the level of single tumor cells (16). This study confirmed that OC cells exhibit upregulation of interferon pathways and metabolism-related genes through epigenetic regulation of promoters. Additionally, through the analysis of gene expression and DNA methylation, they discovered similar patterns within the same lineage between primary and metastatic tumors, providing evidence that metastatic cells may already exist within primary subclones. Subsequently, another research team integrated scRNA-seq and the single-cell assay for transposase-accessible chromatin using sequencing (scATAC-seq) to elucidate the regulatory mechanisms governing cancer cells at a single-cell resolution in ovarian and endometrial tumors (156). This research highlighted the remarkable heterogeneity of gynecologic tumors through the analysis of the relationship between gene expression and chromatin accessibility. Another study innovatively uses single-cell proteomic analysis to characterize brain metastases of OC, revealing possible oncogenic pathways and immunosuppressive mechanisms, which may also be involved in the occurrence of metastases (157). These significant results will pave the way for more individualized therapy choices in OC. Moreover, single-cell genome sequencing has been employed to investigate latent cell-cluster states, focusing on the diversity of copy numbers in ovarian malignancies (158). Single-cell genome sequencing possesses distinct advantages in accurately determining CNAs and mutations (159, 160), thereby enhancing the precision of stratification for treatment and prognosis in genetically unstable tumors (161, 162), which cannot be achieved through transcriptome sequencing alone.

In addition to the methods mentioned above, several other multi-omics approaches have been developed and applied to various diseases. One such method is Co-indexing of transcriptomes and epitopes by sequencing (CITE-seq), which combines the highly multiplexed antibody-based detection of protein markers with unbiased transcriptome profiling for thousands of single cells in parallel (163). This technique is compatible with existing single-cell sequencing approaches and can readily scale as the throughput of these methods increases. CITE-seq has been utilized to uncover spatially distinct germinal center reactions in the tonsil and early immune activation in the skin at the injection site of the Coronavirus Disease 2019 mRNA vaccine (164). Another innovative method is ATAC with Select Antigen Profiling by sequencing (ASAP-seq), which robustly detects cell surface and intracellular proteins using oligo-labeled antibodies in combination with high-throughput single-cell ATAC-seq (165). ASAP-seq allows for the simultaneous profiling of accessible chromatin and protein levels by pairing sparse scATAC-seq data with the robust detection of hundreds of cell surface and intracellular protein markers, with the optional capture of mitochondrial DNA (mtDNA) for clonal tracking. This approach captures three distinct modalities in single cells. In conjunction with DOGMA-seq, a novel adaptation of the CITE-seq method for measuring gene activity across the central dogma of gene regulation, systematic multi-omics profiling has been demonstrated. This approach reveals coordinated and distinct changes in chromatin, RNA, and surface proteins during native hematopoietic differentiation, peripheral blood mononuclear cell stimulation, and as a combinatorial decoder and reporter of multiplexed perturbations in primary T cells. Furthermore, in 2021, 10X Genomics developed a “multiome” approach, which allows for the simultaneous capture of both transcriptomic and epigenomic modalities in the same single cells using Chromium Single Cell Multiome ATAC+Gene Expression (166). This technique eliminates computational errors associated with linking separate datasets and maximizes the information obtained from precious samples. With a unified view of a cell’s open chromatin landscape and gene expression profile, researchers can decipher how cell types and states are established, discover new gene regulatory interactions, and interpret epigenetic profiles alongside key expression markers. Notably, a professional team utilized this approach to investigate the chromatin and gene-regulatory dynamics of the developing human cerebral cortex at single-cell resolution (167). While these new technologies have not yet been widely adopted in ovarian cancer research, it is foreseeable that they will play a significant role in this field in the near future.

These innovative studies utilizing multi-omicss approaches provide insights into crucial genetic and epigenetic characteristics related to OC. Encouragingly, multi-dimensional single-cell sequencing analysis empowers us to acquire precise internal landscapes, detailed profiles, and intercellular interactions within malignant tumors, bringing us closer to unraveling the enigma of cancer initiation and progression. Nonetheless, these emerging sequencing technologies and single-cell-based analysis methods are still in the developmental phase, requiring further experimentation and time to assess their practical applicability.

## 11 Combination of single-cell sequencing with emerging innovative technology

The integration of single-cell sequencing with emerging innovative technologies in OC research holds significant promise as it leverages and synergizes their respective strengths. For instance, a study utilized a 3D culture system to establish an *in vitro* OC model that closely mimics the *in vivo* environment (168). Referred to as single cell-derived metastatic OC spheroids, this 3D system consists of cells derived from metastatic ascites. This model was then integrated with single-cell sequencing to precisely characterize the distinctive features of OC (168). In 2022, a research team integrated multiplex immunofluorescence technology and digital histopathology with single-cell sequencing and whole-genome sequencing to analyze cancer tissues from 42 newly diagnosed OC patients (169). Their findings revealed that tumors with HRD mutations exhibit inflammatory signals, and the immunoediting process is characterized by reduced HLA diversity and impaired CD8+ T cell function. Through the integration of single-cell techniques, this advanced technology holds the promise of enabling detailed research and facilitating more precise treatment strategies for OC.

Meanwhile, the uploading and sharing of single-cell sequencing data maximizes the utilization of scientific research resources. Over the years, numerous studies have compared and analyzed multiple datasets of single-cell sequencing in OC, leading to significant scientific achievements through bioinformatics analysis (170–175). Individual studies based on single-cell sequencing technology face limitations in terms of analysis breadth, scope of investigation, and available data volume. However, these limitations can be mitigated by combining sequencing data from multiple studies, enabling comprehensive analyses that incorporate diverse populations across multiple centers in different countries, thus yielding more representative results. For instance, by conducting a comprehensive analysis of the OC scRNA-seq dataset GSE118828, a research team provided a novel perspective on understanding the progression of OC (176). They successfully identified the predominant M2-like tumor-associated macrophages and discovered several novel markers, including IL4I1, that play a crucial role in tumorigenesis and progression. Building upon these novel findings, they developed a RiskScore system utilizing markers linked to the OC progression that demonstrated a robust correlation with the prognosis of this patient population. Subsequently, a research team performed an integrated analysis of two single-cell datasets and TCGA-OC data, resulting in the identification of a two-gene signature (CXCL13 and IL26) with noteworthy prognostic implications and potential for advancing immunotherapy in OC treatment (177). In 2021, a group carried out an analysis of inter-cellular heterogeneity in OC using scRNA-seq data (178). Through this approach, distinct clusters and their corresponding marker genes were identified. Moreover, through the integration of the TCGA OC dataset, they uncovered a set of differentially expressed marker genes significantly associated with the prognosis of OC, including ANP32E, STAT1, GPRC5A, EGFL6, PMP22, FBXO21, and CYB5R3. Furthermore, single-cell databases play a pivotal role in bioinformatics analysis. For example, a study utilized two single-cell databases (CancerSEA and TISCH) to investigate the function and expression pattern of CD47 in different immune cells at the single-cell level (179). By leveraging these tools, a comprehensive understanding of CD47 and its heterogeneous expression across different immune cell types has been achieved. These findings provide valuable insights into potential biomarkers that can contribute to our understanding of the prognosis and progression of OC.

## 12 Discussion and perspectives

Single-cell sequencing and its derivative technologies have revolutionized our comprehension of OC by facilitating the analysis of individual cells within tumors, unraveling their heterogeneity, and yielding insights into tumor biology and the tumor microenvironment. As previously mentioned, the capture of heterogeneity at the single-cell level has enabled researchers to identify distinct cell populations, elucidate clonal evolution, and unravel the spatial organization of cells within the tumor. Moreover, employing this innovative biological technology, studies have identified diverse stromal cell populations that facilitate tumor growth and impact tumor progression. Investigations into the genetic heterogeneity of OC through single-cell sequencing have unveiled the existence of multiple subpopulations of cancer cells with distinct gene expression profiles and evolutionary trajectories. These recent and innovative insights are vital for comprehending the mechanisms underlying therapy resistance. Furthermore, the combination of single-cell sequencing with spatial transcriptomics enables the revelation of spatial heterogeneity and tumor architecture within ovarian tumors, allowing for the identification of distinct cell populations and their spatial correlations with gene expression patterns.

The application of single-cell sequencing in OC research holds immense promise for advancing diagnostics, therapeutics, and prognostics. By identifying novel cell populations and characterizing their gene expression profiles, new biomarkers for early detection and prognosis of ovarian carcinoma can be potentially revealed. These knowledges may help guide treatment decisions, predict treatment response, and develop personalized therapeutic strategies (180, 181). Additionally, single-cell sequencing has the potential to enhance immunotherapy strategies in OC. By profiling immune cell populations and their functional states within the tumor microenvironment, researchers can identify immunotherapeutic targets and biomarkers of response to immunotherapy, aiding in the development of personalized immunotherapeutic approaches and improvement of patient outcomes.

While single-cell sequencing has brought significant advancements to OC research, several limitations and challenges remain. One major limitation is the technical variability and high costs associated with this novel experiments. Standardizing protocols and optimizing data analysis pipelines are necessary to ensure reproducibility and cost-effectiveness. Another challenge is the limited scalability of single-cell sequencing technologies. The current throughput of single-cell sequencing platforms is often insufficient to comprehensively capture the entire cellular heterogeneity within tumors. Increasing the throughput while maintaining high-quality data will be crucial for more comprehensive analysis. Furthermore, data analysis and interpretation are complex due to the large volume of data generated by single-cell sequencing experiments. Advanced computational algorithms and tools are required for the integration and visualization of multi-omics datasets, enabling the extraction of meaningful biological insights. Based on the aforementioned studies, it is evident that the majority of research focuses on the prevalent EOC, with limited attention given to rare pathological types of OC. In the future, it is crucial to allocate attention to other pathological types as well. Undoubtedly, single-cell sequencing will greatly contribute to the elucidation of the etiology and the development of innovative treatments for OC.

To address the limitations and maximize the potential of single-cell sequencing in OC research, several areas of future development should be considered. Firstly, technological advancements: ongoing improvements in single-cell sequencing technologies, including increased throughput, improved sensitivity, and reduced costs, will enhance the scalability and accessibility of this approach. Secondly, integration of multi-omics data: integrating single-cell sequencing data with other omics data, such as epigenomics and proteomics, will contribute to a more comprehensive understanding of the molecular mechanisms underlying OC. Thirdly, development of computational tools: advancing the development of computational tools and algorithms for data analysis, interpretation, and integration will facilitate the extraction of meaningful insights from complex single-cell datasets. Fourthly, longitudinal studies: conducting longitudinal studies using single-cell sequencing will enable the tracking of clonal evolution and the dynamic changes in cellular populations over time, providing valuable insights into disease progression and treatment response. Lastly, validation and translation: validating single-cell sequencing findings using larger patient cohorts and longitudinal studies is crucial for the clinical translation of research findings. Collaborations among researchers, clinicians, and industry partners will be vital in bridging the gap between research and clinical applications.

In conclusion, single-cell sequencing has revolutionized our understanding of OC by unraveling its heterogeneity, characterizing the tumor microenvironment, and identifying potential biomarkers and therapeutic targets. Despite the existence of challenges and limitations, ongoing advancements in technology, data analysis, and validation efforts offer great promise for the future of single-cell sequencing in enhancing diagnosis, treatment strategies, and patient outcomes in OC.

## Author contributions

ZL: Writing – original draft. HG: Writing – original draft. XX: Writing – original draft. YT: Writing – review & editing. YD: Writing – review & editing. XH: Writing – review & editing.
